# Structural Dynamics of the MscL C-terminal Domain

**DOI:** 10.1038/s41598-017-17396-w

**Published:** 2017-12-08

**Authors:** Navid Bavi, Adam D. Martinac, D. Marien Cortes, Omid Bavi, Pietro Ridone, Takeshi Nomura, Adam P. Hill, Boris Martinac, Eduardo Perozo

**Affiliations:** 1 Victor Chang Cardiac Research Institute, 405 Liverpool Street, Darlinghurst, New South Wales, 2010 Australia; 2 St. Vincent’s Clinical School, The University of New South Wales, Darlinghurst (Sydney), New South Wales, 2010 Australia; 3 School of Mechanical & Mining Engineering, University of Queensland, St. Lucia (Brisbane), QLD 4072 Brisbane, Australia; 4 Texas Tech University Health Sciences Center, Lubbock, Texas 79430 USA; 5 Department of Physics, University of Tehran, Tehran, 1439955961 Iran; 6 Department of Rehabilitation, Kyushu Nutrition Welfare University, Kitakyushu, 800-029 Japan; 7 Department of Biochemistry and Molecular Biology, University of Chicago, 929 E 57th St, Chicago, Illinois 60637 USA

## Abstract

The large conductance mechanosensitive channel (MscL), acts as an osmoprotective emergency valve in bacteria by opening a large, water-filled pore in response to changes in membrane tension. In its closed configuration, the last 36 residues at the C-terminus form a bundle of five α-helices co-linear with the five-fold axis of symmetry. Here, we examined the structural dynamics of the C-terminus of EcMscL using site-directed spin labelling electron paramagnetic resonance (SDSL EPR) spectroscopy. These experiments were complemented with computational modelling including molecular dynamics (MD) simulations and finite element (FE) modelling. Our results show that under physiological conditions, the C-terminus is indeed an α-helical bundle, located near the five-fold symmetry axis of the molecule. Both experiments and computational modelling demonstrate that only the top part of the C-terminal domain (from the residue A110 to E118) dissociates during the channel gating, while the rest of the C-terminus stays assembled. This result is consistent with the view that the C-terminus functions as a molecular sieve and stabilizer of the oligomeric MscL structure as previously suggested.

## Introduction

The bacterial mechanosensitive channel of large conductance (MscL), has become a prototype molecular system in the study of mechanotransduction and its evolutionary origins^1,2^. The three-dimensional (3D) structure of MscL from *Mycobacterium tuberculosis* (MtMscL) in its closed conformation was resolved at 3.5 Å^3^. The channel is a homopentamer consisting of N- and C-terminal domains facing the cytoplasm and TM1 and TM2 transmembrane helices connected by a periplasmic loop^3–6^. The closed channel structure was also determined in the lipid bilayer by site-directed spin labelling electron paramagnetic resonance (SDSL EPR) spectroscopy^7^. Moreover, the open channel structure was also determined by SDSL EPR spectroscopy^8^ allowing for estimation of the size of the open channel pore in a very good agreement with electrophysiological sieving studies^9^. Later on, an improved open *E*. *coli* MscL (EcMscL) 3D structure was determined using both ensemble and single molecule site-directed fluorofore labelling (SDFL) FRET spectroscopy in combination with MD simulations^6,10,11^ in agreement with the X-ray structure of an expanded form of an archaeal MscL homolog from *Methanosarcina acetivorans* ^12^. While the overall gating-related structural changes in MscL have largely been established^8,11,13,14^ the structural dynamics and physiological role of the C-terminal domain has thus far been controversial. Several studies have suggested that the C-terminus should remain intact during gating and could function as a molecular sieve^15,16^, while others suggested that this helical bundle should actively participate in gating and that opening of the MscL channel was accompanied by complete dissociation of the bundle^13,17^. More recently, the X-ray structure of the EcMscL C-terminus domain was obtained at 1.45 Å^18^, suggesting that even in the absence of anchoring biases by the TM segments, the bundle stays oligomerized.

To address the question of the C-terminus structural dynamics during MscL gating we used SDSL EPR spectroscopy to characterize the conformations of the EcMscL C-terminal bundle under conditions that stabilize either the closed or the open channel conformations. Residue mobility and environmental parameters obtained by SDSL EPR spectroscopy were used to compare its conformation in the membrane with that of the crystal structure and alternative packing models. In addition, we used molecular dynamics (MD) simulations and finite element (FE) modelling to examine the role of polar and electrostatic interactions in maintaining the stability of the MscL C-terminal bundle in different physical environments. Our experimental and computational results indicate that the bundle stretches apart only at its upper half while the central and the bundle end remain intact, consistent with its role as a molecular sieve and stabilizer of the MscL oligomeric structure.

## Materials and Methods

### Expression, labelling and liposome reconstitution of the MscL protein

27 C-terminal residues, from Ala 110 to Ser 136 were mutated to cysteine for spin labelling. Mutagenesis was performed by oligonucleotide mismatch site-directed mutagenesis using the Transformer kit (CLONTECH Laboratories, Inc.) and confirmed by dideoxy DNA sequencing. Each of the 27 MscL mutants were cloned into pQE32 vector (N-terminal 6xHis tag) and expressed in *E*. *coli*, followed by MscL protein solubilization using dodecylmaltoside (DDM). Mutant channels were purified using Co^2+^-based resin (Talon resin; CLONTECH Laboratories, Inc., as described previously^7^) and spin labelled overnight with methanethiosulfonate spin label (Toronto Research) at a 10:1 label/channel molar ratio and reconstituted at a 500:1 lipid/channel molar ratio by dilution in PBS.

### EPR spectroscopy and data analysis

EPR spectroscopy was performed as described previously^7,19^. Briefly, X-band CW EPR spectra were obtained in a Bruker EMX spectrometer fitted with a dielectric resonator at 2-mW incident power, 100 kHz modulation frequency and 1 G modulation amplitude. Solvent accessibility was estimated by determining the collision frequency of a given nitroxide spin label with the paramagnetic complex Ni(II)ethylenediaminediacetate (Ni-EddA). Ni-EddA accessibility was derived from Power saturation curves, obtained for each spin-labelled mutant after equilibration in N_2_ as control and N_2_ in the presence of 200 mM Ni-Edda as relaxing agents. All EPR data were obtained at room temperature from MscL mutants expressed in *E*. *coli*, purified by Co^2+^-based resin, spin labelled and reconstituted into monounsaturated 18:1 phosphatidylcholine liposomes (POPC) (Avanti Polar Lipids, Inc., Alabaster, Alabama) at a protein-to-lipid molar ratio of 1:500^20^. Expression levels of all cysteine mutants analysed in this study were almost identical to that of the wild-type MscL protein.

### Electrophysiological recordings

Spin-labelled channel mutants were tested for function and activation by lysophosphatidylcholine (LPC) 12:0 in patch-clamp analysis. For illustration, current traces recorded from the V120C mutant channels reconstituted into liposomes made of azolectin or POPC (18:1) are shown in Fig. 5. The channel was activated in azolectin liposomes by negative pressure applied to the patch pipette (Fig. 5A) as well as by the addition of LPC to excised POPC liposome patches (Fig. 5B and C). Activation by membrane tension and LPC of the wild-type and various cysteine MscL mutant channels used for SDSL EPR^20^ and SDFL FRET^13^ spectroscopic studies was previously reported and independently confirmed. Fully and partially activated channels were recorded from approximately 53% of patches (n = 19). Typically, spontaneous channel gating occurred between 2–10 min after addition of LPC^20,21^. In this study, the application of LPC to POPC liposomes activated V120C channels in approximately 70% of the patches (n = 12). Activity of the MscL mutants was recorded from bilayer blisters in symmetrical solutions (200 mM KCl, 40 mM MgCl_2_ and 5 mM HEPES buffer, pH 7.2/KOH) upon application of negative hydrostatic pressure to the patch pipette, as previously reported^22^. The 1:500 protein-to-lipid ratio by weight was used in all the patch-clamp electrophysiology experiments^22^.

### Molecular dynamics simulations

The 3D model of EcMscL was generated based on the crystal structure of the MscL homolog of *M*. *tuberculosis* (PDB ID: 2OAR) and the crystal structure of *E*. *coli* C-terminus (PDB ID: 4LKU) using Phyre2^23^ and Swiss-Model^24^. The MscL model was embedded into a POPE (1-palmitoyl-2-oleoyl-sn-glycero-3-phosphoethanolamine) lipid bilayer. The protein and lipids were next solvated with TIP3P water box and 200 mM KCl. The system was equilibrated for 62 ns with a time-step of 2 fs with no restraints. After equilibration, to see the channel opening, a 75 mN/m surface tension was applied on the lipid molecules for 200 ns in an NγP_z_T ensemble. A further 25 mN/m was added to the previous surface tension (100 mN/m in total) and applied to the membrane for another 6 ns (268 ns in total). All simulations were performed with the NAMD 2–10 package, where CHARMM36 Force Field was employed. Visual Molecular Dynamics (VMD)^25^ and Pymol were used for all visualizations.

### Finite Element Modelling

Finite Element (FE) analysis was applied to enable modelling of the MscL C-terminal structural dynamics at the continuum level. A 3D MscL model embedded in the lipid bilayer was constructed using ABAQUS (ABAQUS FEA. Simulia, Providence, RI 02909-2499, USA) by generating data points in space based on the α-carbon coordinates obtained from the 3D crystal structure of MscL^4,26^. These points do not have any properties other than position. The lipid bilayer was assumed to behave as a linear elastic plate^27,28^, and the secondary structural elements making the MscL protein were treated as elastic cylinders/rods, as described^6,29,30^. The Young’s modulus of the bilayer was assumed to be 4.3 MPa as previously determined using excised patch fluorometry^28^ and the Young’s moduli of the MscL N-terminal (0.35 GPa), TM1 (2.6 GPa), TM2 (3.4 GPa) and C-terminal (7.7 GPa) α-helices were determined using steered molecular dynamics (MD) simulations^31^. The dimensions of the rods were: radius r = 2.5 Å, the lengths, N-terminus = 18.65 Å, TM1 = 47.33 Å, TM2 = 42.51 Å, C-terminus = 36.06 Å. The distance between the MscL channel boundary and the lipid bilayer, in the model, was not greater than 2.5–3.0 Å. So that no relative motion could occur, these two separate entities were bound together using a tie constraint, effectively emulating the N-terminal and TM2 helix, and the tip/head of the TM1 helix being embedded in the lipid bilayer. Tetrahedral elements were used for meshing the lipid bilayer and the TM1 and TM2 helices. To simulate the physiologically plausible deformation of the MscL channel the rods in our model were hybrid incompressible by assuming the Poisson’s ratio to be 0.48 (almost incompressible^31,32^).

The major interactions between the five helices of the C-terminal domain are the three distinct networks of electrostatic interactions which are located at the top, middle and bottom of the channel. The top and bottom electrostatic interactions can be considered to be electrostatic “caps” which are stabilized by hydrogen bond networks^16^. In order to account for all hydrogen bonds encountered between the atoms in the C-terminal helix bundle residues the DSSP approximation was used^23^, an algorithm that assigns secondary structure to amino acids in a protein and can be used for estimation of hydrogen bond energy (SI Eq. 3–4). The central electrostatic interactions encompass the circumference of the C-terminal bundle creating a “belt” like structure around the coiled coil^18^. This central belt contains an electrostatic salt bridge formed by the residues E124, D127 and R126 below the RKKEE charged amino acid cluster^33^. To determine the electrostatic and polar forces acting in the 3D MscL FE model in an electrolyte solution the Debye-Hückel relation was used (SI Eq. 5–7).

The electrostatic and polar interactions were implemented in the model by using connector elements which allow for a nonlinear elastic translation of force. When set up as an elastic translating element, the connectors permitted the input of a force value with a corresponding displacement value, creating a table of data points which emulate a nonlinear force in the constitutive space. The table of data used for each interaction was calculated from the equations which govern the respective interaction behaviours (SI Eq. 1–7). The positions where the connectors were applied are based on the crystallographic sites of the respective interacting amino acid residues (Fig. S3).

## Results

**Figure 1:**
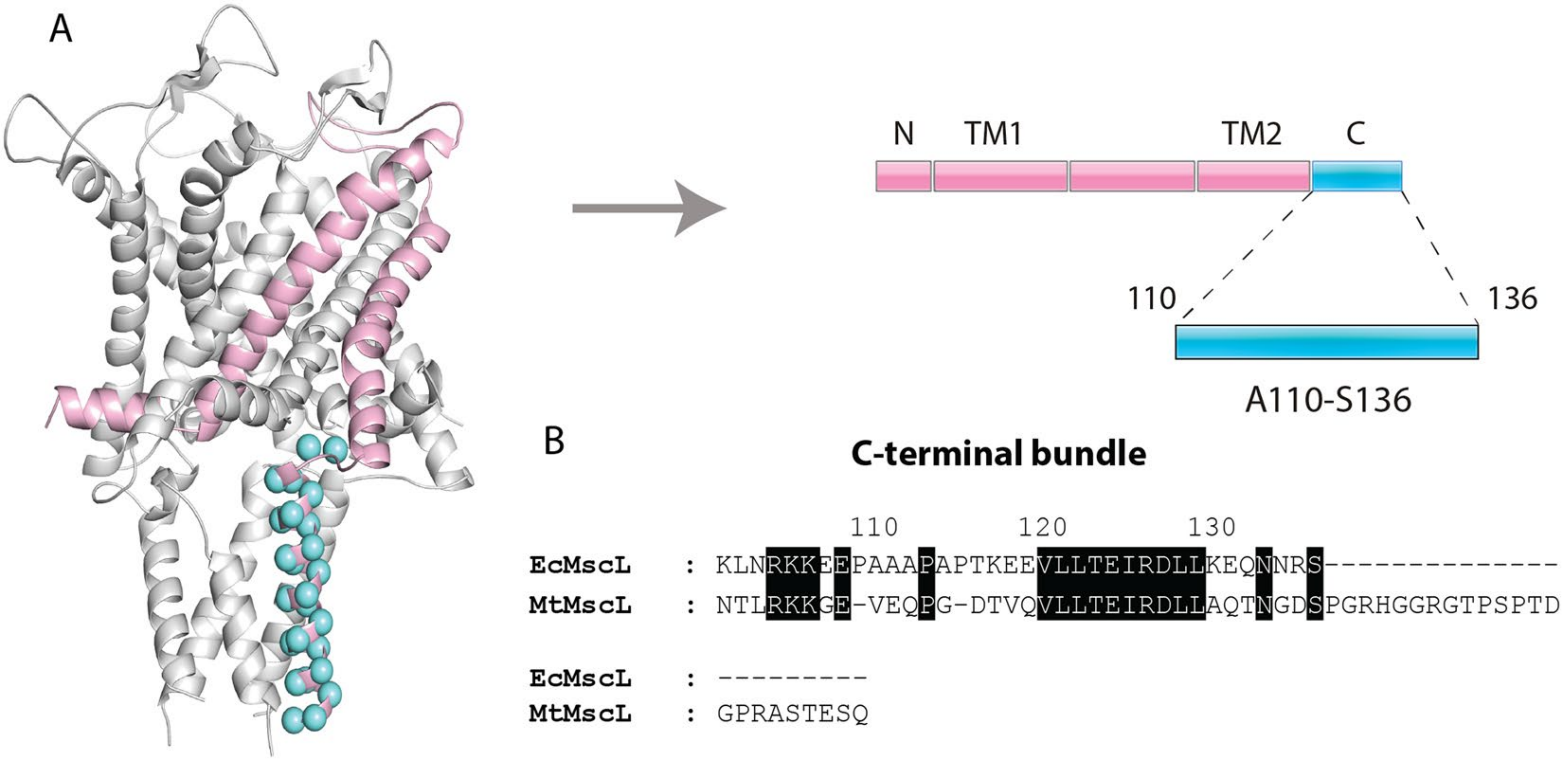
3D homology model of the closed EcMscL channel. (**A**) Single MscL subunit (rose pink) showing amino acid residues comprising the A110-S136 segment of the C-terminal bundle encompassing 27 residues subjected to cysteine scanning mutagenesis (blue spheres). The single MscL monomer is represented as a part of the channel pentamer according to the 3D homology model of EcMscL as described in the methods^6^ (left). Linear representation of the membrane topology of EcMscL (right). The N- and C-terminal domains, transmembrane helices TM1 and TM2 and the periplasmic and cytoplasmic loops are represented by rose pink and blue rectangles, respectively. (**B**) Sequence alignment of the C-terminal bundle of EcMscL and MtMscL. Identical residues are highlighted in black. The numerical equivalence between the residues shown in the figure was generated using a ClustalW pairwise sequence alignment between the two helical bundle sequences.

A single EcMscL monomer (rose pink) highlighted in the 3D homology structure of EcMscL (Fig. 1A) contains 27 amino acid residues (shown as cyan spheres) comprising the A110-S136 segment of the C-terminal helical bundle. Structural comparison between the helical bundles of EcMscL and MtMscL required a residue adjustment between the two MscL homologues by aligning their corresponding sequences (Fig. 1B). There is 48% identity between the primary amino acid sequence of the EcMscL and MtMscL C-terminal segment in addition to the difference in their total number of residues (136 and 151, respectively). All 27 residues in EcMscL helical bundle were subjected to cysteine scanning mutagenesis, spin labelling and EPR spectroscopic analysis.

**Figure 2:**
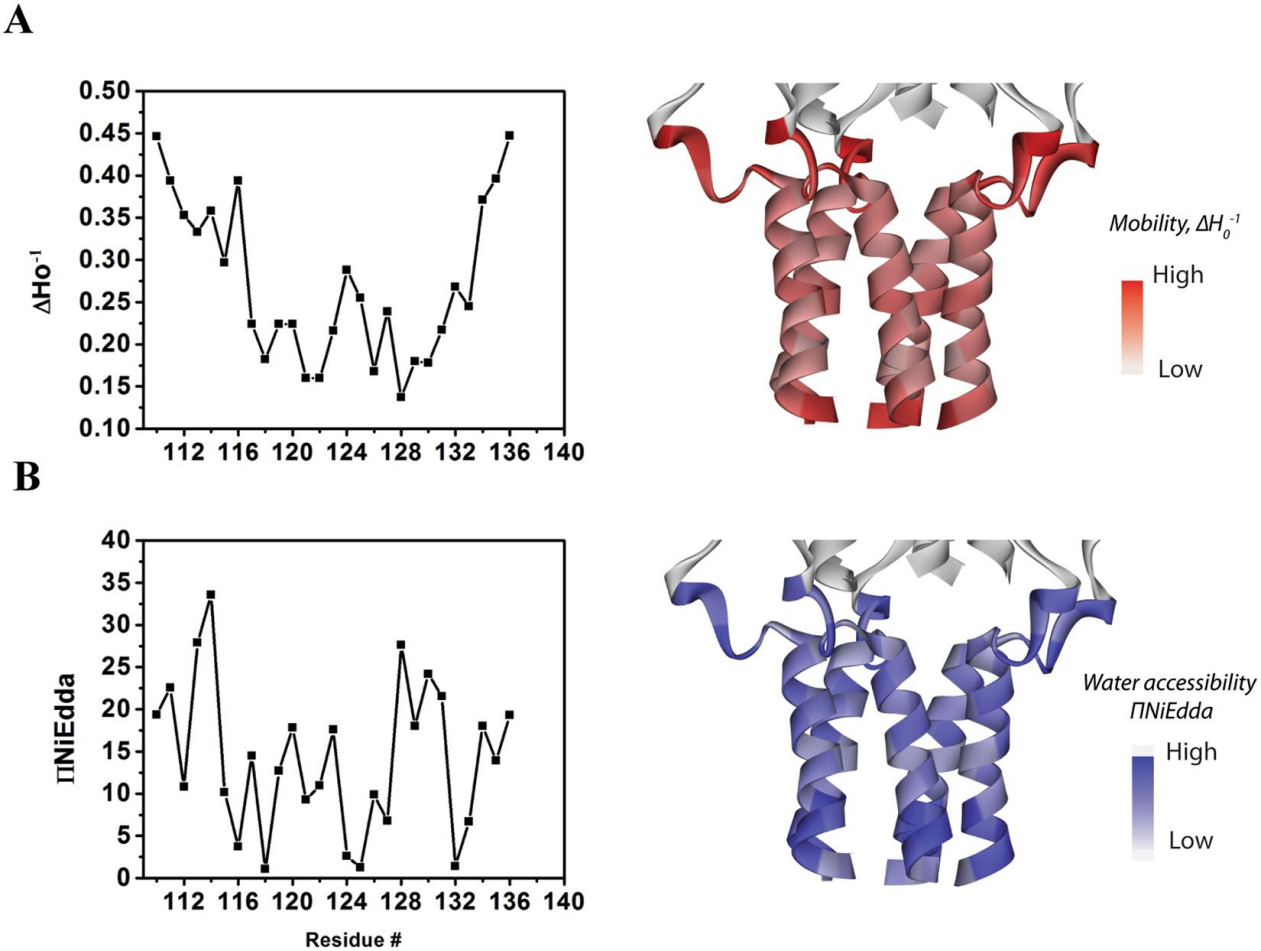
Residue-specific environmental parameter profiles obtained for the A110–S136 segment of the C-terminal bundle. (**A**) Mobility parameter ΔH_0_^–1^ indicates the greatest mobility for the first seven residues A110–T116 and three last residues N134–S136, which suggests these residues form a free loop and have no defined secondary structure, respectively (left). A periodicity in the mobility of the spin label from T116 through to E131 residues is consistent with the observed α-helical structure of this portion of the C-terminal bundle (right) in the crystal structure of MtMscL (D108–Q123 in MtMscL), which is not considered to be in the fully open state but an expanded state (Li, Jie, *et al*., 2015, PNAS). Importantly however, MaMscL has a vastly different C-terminus compared to the EcMscL in terms of the number of helices, sequence and structure. Nevertheless, the transmembrane helices share high similarity in sequence and gating mechanism^6^. (**B**) NiEdda accessibility parameter ΠNiEdda shows high accessibility of most helical bundle residues to NiEdda and thus to the aqueous compartment consistent with the cytoplasmic location of the bundle (left). The profile of ΠNiEdda is also roughly correlates to the profile of the mobility parameter ΔH_0_^−1^ shown in (**A**). Accessibility to NiEdda indicated in blue on the crystal structure of the helical bundle (right).

The residue-specific environmental parameter profiles for the complete set of the 27 C-terminal residues in the closed configuration of the MscL channel are shown in Fig. 2. The mobility parameter ΔH_0_^−1^, derived from the inverse of the width of the central resonance line^34^, was obtained for each of the 27 residues comprising the A110-S136 segment of the C-terminal bundle (Fig. 2A). The first seven residues (A110 – T116) as well as the last three residues (N134–S136) exhibited high mobility indicating that these residues form a loop or have no defined secondary structure, respectively, in the segment in accordance with the 3D crystal structure of MscL (Fig. 1A). The spin labels of the remaining 16 residues (T116–E131) are more dynamically restricted, indicating that they are constrained within secondary structure elements in this portion of the helical bundle indicated by the X-ray crystal structure.

Solvent accessibilities for each of the 27 helical bundle residues were estimated from power saturation experiments performed in the presence of a water-soluble Ni^2+^ chelate complex (NiEdda) as previously described^20^. The profile of the ΠNiEdda accessibility parameter (Fig. 2B) suggests high water accessibility for majority of the bundle residues, as expected from a region of the protein fully outside of the membrane. In spite of some key differences, the overall similarity of the profile of ΠNiEdda to the profile obtained for the mobility parameter ΔH_0_^–1^ is evident, both of which are consistent with the 3D structure of MtMscL and the cytoplasmic location of the helical bundle.

**Figure 3:**
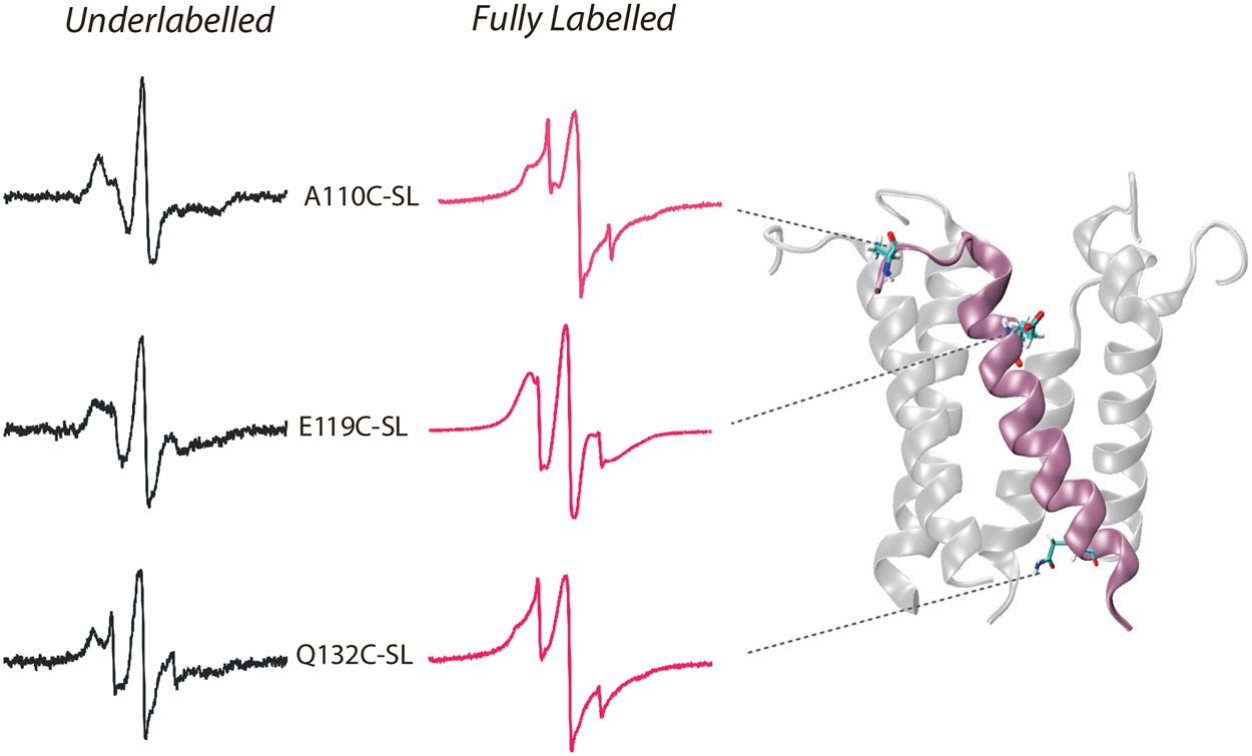
Examples of EPR spectra of the C-terminal residues of EcMscL. X-band EPR spectra of underlabelled and fully spin-labelled A110C, E119C and Q132C mutants showing increased spin mobility at the top and the bottom of the A110–S136 segment of the helical bundle and reduced mobility in the middle of the A110–S136 segment. All spectra were obtained using loop-gap resonator with the microwave power of 2 mW and field modulation of 1 G.

In a pentameric system like MscL, spin-spin interactions arise from same-residue inter-subunit interactions near the axis of symmetry. These interactions disappear when the channel is under-labelled so that on average, there is only one spin label per channel. A comparison of spectra from selected residues that have been either fully spin-labelled or under-labelled at a 1:10 ratio spin-label:protein (black traces) is shown in Fig. 3.

The presence of spectral broadening due to dipolar coupling is evident for residues A110 at the top and Q132 at the bottom part of the helical bundle (both are also highly mobile). On the other hand, residue E119 in the centre of the helix displays dipolar broadening but shows restricted mobility due to proximity of the residues near the centre of the helical bundle, as clearly indicated by the comparison of amplitudes and broadening of the low field and the high field lines. Coupled with the environmental parameter profile (see Fig. 2), these results are reflection of the helical bundle formed by the A110–S136 segment and seen in the MscL crystal structure.

**Figure 4:**
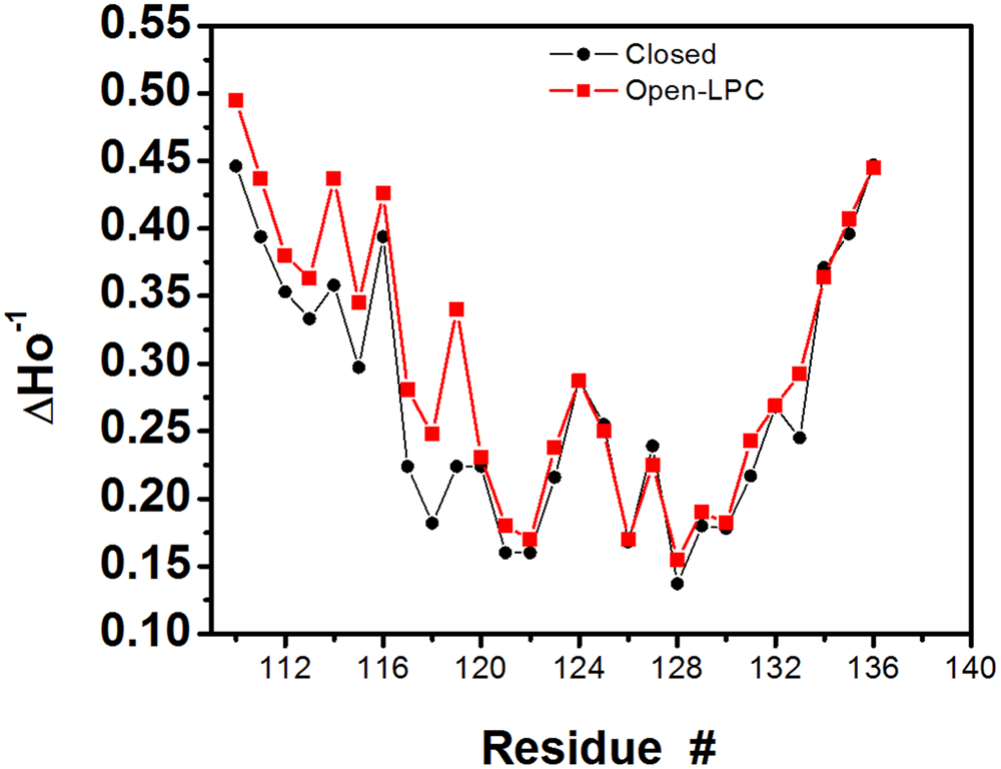
Mobility parameter ΔH_0_^−1^ profiles of the C-terminal helical bundle. The mobility parameter ΔH_0_^−1^ was obtained in the closed (black) and open (red) configuration of MscL. The spin-labelled mutant channels were opened by applying 25 mol% LPC to liposomes reconstituted with the mutant proteins, as previously reported^6,20^. Except for the first ten amino acid residues (A110 through E119) showing higher mobility in the open channel configuration compared to the closed channel, the profiles are almost identical showing no difference in the mobility of the remaining 17 residues (V120 through S136) between the closed and the open MscL channel.

Next, each helical bundle mutant channel was examined by EPR spectroscopy under conditions that stabilized the open conformation of the channel. We have reported previously that distortion of transbilayer pressure profiles through the use of mixtures of lipids with different geometries leads to open MscL channels in patch-clamp experiments with no suction applied to the patch pipette^6,8,20,35^. A comparison of EPR results obtained from closed versus open channels (in the absence and the presence of 25% molar LPC respectively) is shown in Fig. 4.

**Figure 5:**
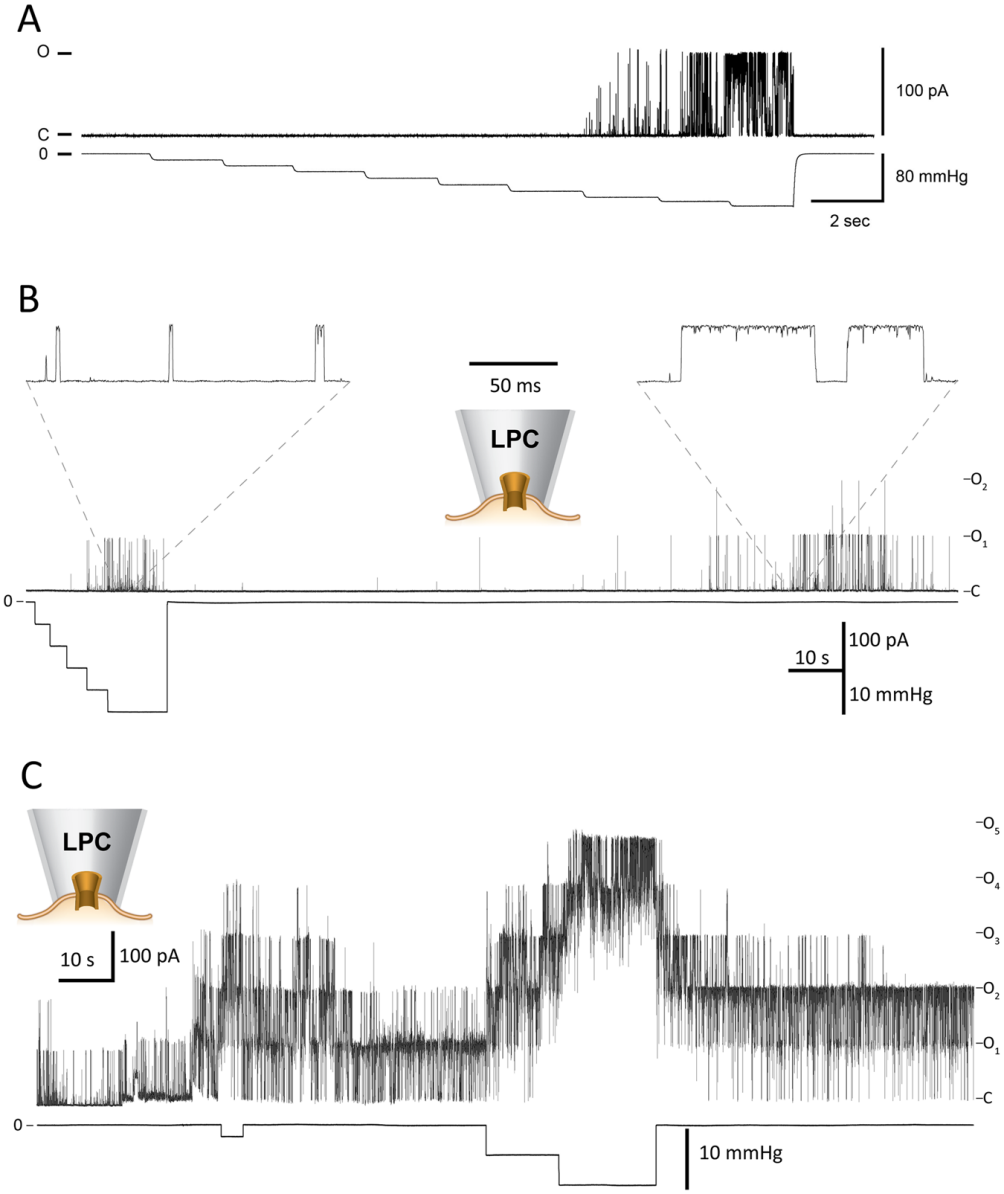
Patch-clamp recording from purified MscL mutant V120C reconstituted into liposomes. (**A**) The MscL-V120C mutant current recorded upon the application of 10 mmHg suction steps onto the patch area. The channel currents (top) and the negative pressure applied to the inside-out azolectin liposome patch through the patch pipette (bottom) are shown. (**B**) Activation of the mutant channel by application of 3 μM LPC in the patch pipette, which is much less than 25 mol% used in EPR experiments (Fig. 4). (Note that it took ∼2 min for LPC to diffuse inside the pipette to reach and incorporate in the monounsaturated (18:1) POPC liposome patch and activate the channel.) The channel activity is characterized by brief channel openings. Expanded views show the openings of the MscL-V120C mutant in the presence (left) and absence of suction (right) after LPC incorporation into the liposome patch. (**C**) The activation of the MscL mutant channel in the presence of 5 μM LPC in the patch pipette before, during and after application of suction to the pipette. Note longer openings of multiple channels when compared to 3 μM LPC recordings. Pipette potential was +30 mV.

The MscL mutants could also be activated in patch clamp experiments by the addition of LPC to the excised liposome patch (Fig. 5B), as shown previously in several studies^11,20,21^. The addition of LPC in the presence of negative pressure (suction) applied to the patch pipette produces a significant increase in the MscL-V120C channel activity and once the suction is released the channels remain constitutively open due to the asymmetry in the lateral pressure profile induced by LPC favouring open state of the channel (Fig. 5C)^20^. Importantly, compared to pure POPC higher concentrations of LPC were required (up to ∼300 μM) to activate the MscL-V120C mutant channel in azolectin liposomes (SI Fig. S1). This is likely due to the fact that azolectin consists of a mixture of phospholipids (extracted from soy bean), which can reduce the effect of LPC.

While the mobility of the spin labels for residues V120–S136 is almost identical between the two states, there is small but significant increase in residue mobility between residues A110 and E119. Therefore, we suggest that during the opening of MscL only a subset of residues at the top of the helical bundle (A110–E119) are moving away from the fivefold symmetry axis, whereas the majority remains in a similar configuration as in the closed channel.

**Figure 6:**
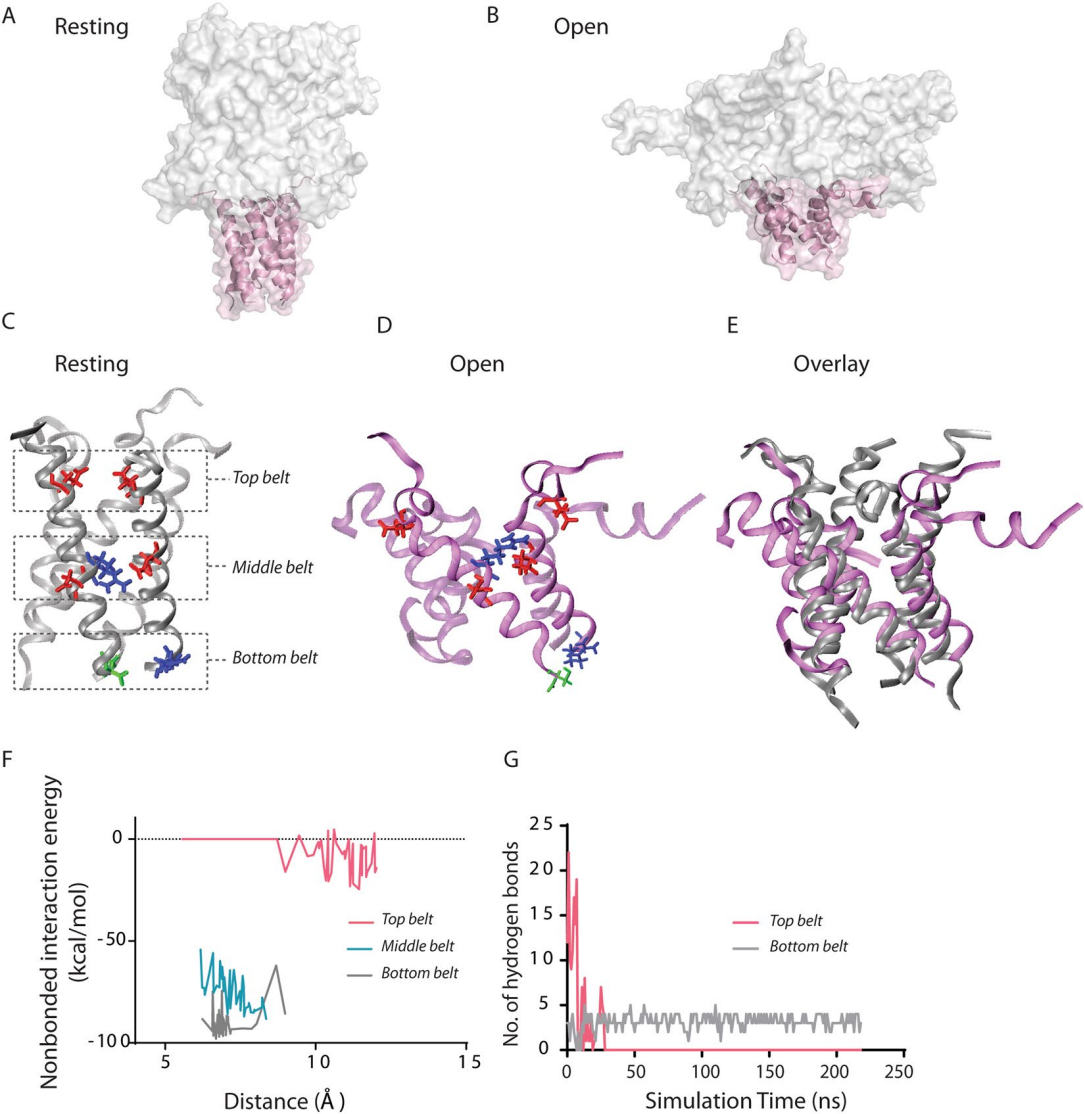
Conformations of the MscL C-terminal bundle obtained during MscL gating using all-atom molecular dynamics simulations. EcMscL structure (top panel) showing the helical bundle in the resting (closed) state after the initial relaxation of ∼60 ns. (**A**) and in the open state (**B**). The helical bundle is coloured in rose pink. The close-up perspective of the C-terminal bundle (**C**) at rest, (**D**) at the open state and (**E**) the overlay of the two states. Top, middle and bottom interactions between the residues E (red), R (blue), D (red) and S (green). (**F**) Change in the total interaction energy during the channel gating is plotted against the alpha-carbon to alpha-carbon distance of the residues forming the top, middle and bottom belts. The middle belt residue interaction is electrostatic due to the salt bridge formation between E124 and R126 and D127 residues of the adjacent subunit. A tight and stable hydrogen bond also exists between R135 and S136 of the adjacent subunit (bottom belt). (**G**) The number of hydrogen bonds in the top belt decreases dramatically from ~ 20 to zero during the channel opening, while the number of hydrogen bonds in the bottom belt remains stable. For all the hydrogen bond calculations, we used a donor-acceptor distance of 3.5 Å and angle cut-off of 30°. Overall, the figure shows low interaction energy in the top belt and high interaction energy in the middle belt. The helical bundle dissociates during MscL opening around the top part but remains intact in the middle and bottom parts during 210 ns simulation time (206 ns of the stretching simulation plus 4 ns of the equilibration step; therefore, 206 + 4 = 210 ns).

Using all-atom molecular dynamics (MD) simulations, we examined the structural dynamics of the A110–S136 segment during gating of EcMscL (Fig. 6). The MD results indicated that in the closed state in a POPE bilayer, most of the A110–S136 segment forms a stable helical bundle (Fig. 6A). Upon channel opening, the first ten residues of the A110–S136 segment (A110 to E119) dissociates and bends outwardly while the rest of the helical bundle remains intact (Fig. 6B). Dissociation also occurs in the top belt (E118) of the A110–S136 segment, which in the closed state are kept together by hydrogen bonds with water molecules (Fig. 6B)^8^. We monitored the prevailing interaction energies dependent on the inter-helical distances in the A110–S136 segment including top, middle and electrostatic belts (Fig. 6C). The interaction energy between the helices at the top belt (E118) was very low compared to those at the middle electrostatic belt (E124, R126 and D127) and bottom belt (R135 and S136). Interaction energies in the middle and bottom belts were high and stayed relatively stable during gating, resulting in less outward helical movement/deformations in this region of the helical bundle. Further, while the number of hydrogen bonds in the upper belt between the E118 residues and the water molecules decreased dramatically upon channel opening, the number of hydrogen bonds between the R135 and S136 in the middle belt was unchanged during gating (Fig. 6D).

**Figure 7:**
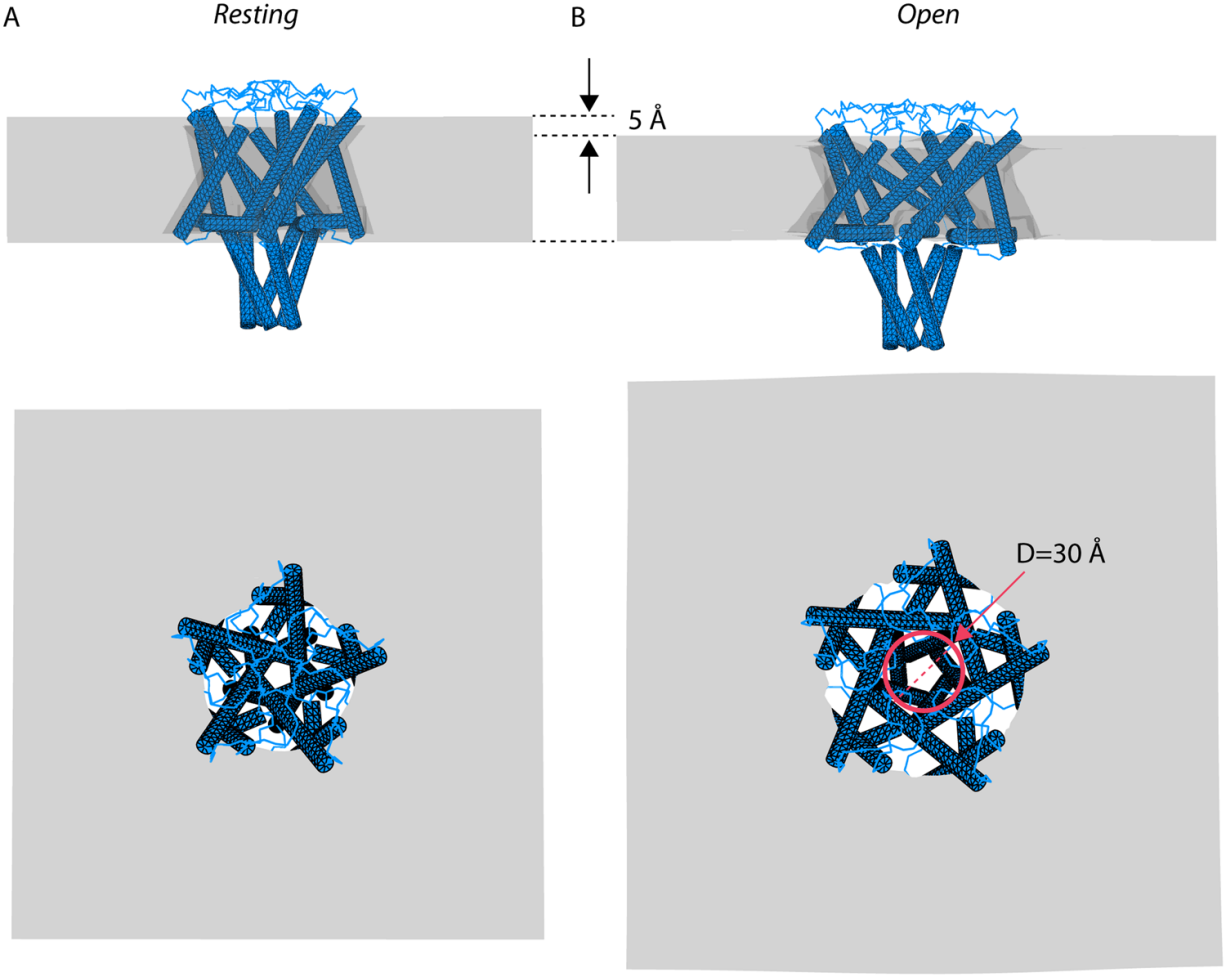
C-terminal helical bundle structural dynamics using finite element (FE) simulation for water-salt (KCl) environment. (**A**) Side and top views of the closed state and (**B**) open state of MscL are shown. In the open state (where the pore diameter is D ∼ 30 Å and the membrane thins from 35 Å to ~30 Å) there is outward bending in the upper part of the helical bundle compared to the resting (equilibrated) state, while the rest of the bundle does not change during the channel opening.

In order to assess the macromolecular consequences of these changes in hydrogen bonding, we next used finite element (FE) (continuum) modelling to monitor the dynamic conformation of the helical bundle during channel gating in different physical environments including vacuum (Fig. S4), water (Fig. S5) and water-salt (0.3 M KCl) (Fig. 7).The aim was to investigate whether the forces affecting C-terminus dynamics were comparable to those predicted from the MD simulations and experimental EPR results. This was done by developing a continuum model and applying the predicted forces while leaving out the detailed molecular interactions (details of the FE calculations are included in the **SI**)^6,34,36,37^. This method has recently been adopted successfully for computational study of several proteins including MscL^34,38^. Our FE model was based on the crystallographic coordinates of the 3D structure of MscL^4,18^ (see Materials and Methods).

A simulated membrane tension of 9.5 mN/m (2.63 MPa)^6,37^ was applied to open the channel in three environments - vacuum, water and salt-water (Figs S4 and S5, Fig. 7). Changes in the macromolecular structure of both the bilayer and the transmembrane region of MscL showed close agreement with experimental values (SI Table S1). The side view of the open channel configuration (Fig. 7) suggests that the bundle helices (in natural salt environment) do not dissociate from each other; only the upper part of the bundle bends outwardly slightly. Interestingly, the results of the simulation for MscL in vacuum show that after opening of the channel, the tilt of the helical bundle is considerably more pronounced as water mediated hydrogen bonding is absent in the top belt interactions (Fig. S4), suggesting that hydrogen bonding is important for the structural stability of the bundle.

## Discussion

MscL is gated by bilayer tension and functions as an electromechanical switch converting membrane bilayer deformations into concerted protein motion^39,40^. By opening a large non-selective ion channel pore of ~30 Å in diameter^8,9,11,13^ that allows unhindered permeation of solvent and solutes on a millisecond time scale, the channel functions as a bacterial safety valve that opens during hypo-osmotic shocks due to changes in environmental osmolarity^41–43^. The C-terminal bundle of EcMscL (from residue A110 to S136) has been shown not to participate in MscL gating and has thus been proposed to have a function of a size-exclusion filter preventing loss of essential large (>6.5kDa^38^) metabolites during MscL opening^13,39^. Furthermore, the Δ110-MscL deletion mutant lacking the A110-S136 segment of the helical bundle was shown to be functional and mechanosensitive, although it exhibited activity characterized by frequent gating at subconducting levels compared to the wild-type channel^44^ suggesting that the C-terminal bundle may also be required for the formation and stabilization of the pentameric structure of the channel^43^. According to these studies the C-terminal domain is stably associated in both closed and open states of the MscL channel. Others have suggested that the stability of the domain was pH dependent^33^, which added another dimension to the function of this domain, namely that of a pH-sensor.

However, this view that the C-terminal bundle is stable during gating has recently been challenged. In an electron microscopy study using low-angle rotary shadowing^17^ the open pore of the gain-of-function (GOF) mutant G22N of MscL was shown to comprise both the transmembrane TM1 and C-terminal helices. According to this study the opening of MscL should be accompanied by dissociation of the helical bundle during the opening of the channel pore^17^.

To resolve which structural model is correct and to understand the structural changes within the MscL helical bundle during the gating cycle of the channel, we have used a combination of site-directed EPR spectroscopy and computational modelling (MD and FE simulations) for residues A110 to S136 of EcMscL. The motional information contained in the nitroxide EPR spectral line shape together with the results of standard collisional relaxation methods were used to determine the extent of residue accessibility to NiEdda in closed and open MscL channels. The experimental EPR data (Fig. 4) together with the computational MD simulations, and FE modelling (Figs 6 and 7) point to a gating mechanism where the C-terminal bundle only partially dissociates during MscL opening. This partial dissociation is a consequence of a slight outward bending of the bundle helices (Fig. 6B), so that several residues close to the cytoplasmic loop (A110–E119) separate due to the rotation and tilting of the TM2 helix^8^. Most obvious change in mobility was observed at residue E118 (Fig. 4) due to breakage of hydrogen bonds between subunits in this region (Fig. 6G). This is as a result of weak and unstable hydrogen bonds mediated by water molecules in this region (top belt).

**Figure 8:**
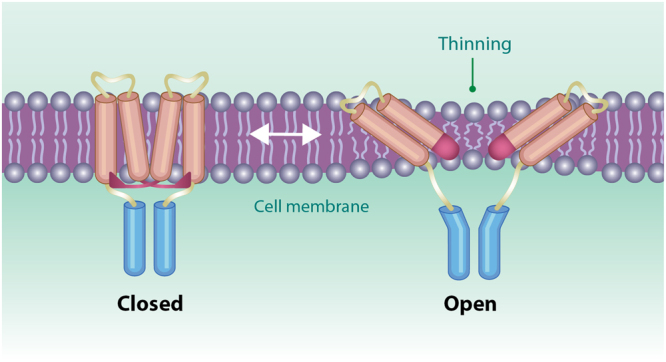
A diagram showing the main structural conformations during MscL opening. The C-terminal bundle remains largely intact during MscL structural change from the closed to the open state. The model also shows the MscL N-terminal domain (red rectangle) fusing with the TM1 transmembrane helix to form a single continuous α-helix in the fully open channel^6,47^.

In contrast, residues further along the bundle sequence (V120–S136) remain assembled as in the closed configuration of the channel (Figs 4, 6 and 7). This association is favoured by high inter-helical electrostatic (middle belt) and hydrogen bonding (bottom belt) interactions in this region. Our model schematically depicted in Fig. 8, is also in good agreement with a recent FRET spectroscopic study suggesting that the top of the C-terminal bundle separates to create a larger entrance to the pore, allowing molecules such as streptomycin to pass through the channel^45^. However, the rest of the helical bundle end remains associated during the gating cycle and likely functions both as a molecular sieve and as a stabilizer of the MscL oligomeric structure^15–17,46^. As such, the C-terminal bundle of MscL helps prevent the escape of essential cytosolic components larger than 6.5 kDa during hypoosmotic shock.

## Electronic supplementary material

Supplementary information
