# Supplementary material for: Structural Dynamics of the MscL C-terminal Domain
Source: Sci Rep. 2017 Dec 8;7:17229. doi: 10.1038/s41598-017-17396-w (PMC5722894; doi:10.1038/s41598-017-17396-w)
Supplement: Supplementary file 1 — Supplementary information [file 41598_2017_17396_MOESM1_ESM.docx]

**Supplementary Information**

**Structural Dynamics of the MscL C-terminal Domain**

Navid Bavi^1,2,*^, Adam D. Martinac^1,3,*^, Marien D. Cortes^4^, Omid Bavi^1,5^, Pietro Ridone^1,2^, Takeshi Nomura^1,6^, Adam P. Hill^1,2^, Boris Martinac^1,2,#^, and Eduardo Perozo^7,#^

^1^Victor Chang Cardiac Research Institute, 405 Liverpool Street, Darlinghurst, New South Wales 2010, Australia,

^2^St. Vincent’s Clinical School, The University of New South Wales, Darlinghurst (Sydney), New South Wales 2010, Australia,

^3^School of Mechanical & Mining Engineering, University of Queensland, St. Lucia (Brisbane), QLD 4072, Australia,

^4^Texas Tech University Health Sciences Center, Lubbock, Texas 79430, USA (M.D.C.), ^5^Department of Physics, University of Tehran, Tehran 1439955961, Iran.

^6^Department of Rehabilitation, Kyushu Nutrition Welfare University, Kitakyushu 800-029, Japan,

^7^Department of Biochemistry and Molecular Biology, University of Chicago, 929 E 57^th^ St, Chicago, Illinois 60637, USA.

***Electrophysiological recordings***

In **Fig. S1** 300 μM LPC was added to the patch pipette to activate V120C MscL mutant reconstituted into azolectin liposomes. We used LPC concentrations much lower than 25 mol% to observe channel activation in pure POPC liposomes (**Fig. 5**) and prevent patch breaking, because during the patch clamp experiments liposome patches are under residual stress due to the gigaohm seal formation, which causes the bilayer to break easier in the presence of LPC^1^. Under EPR measurement conditions the liposome bilayer is not under basal stress and can tolerate much higher LPC concentrations (>25 mol% LPC with POPC). Note that contrary to the EPR liposome experiments, it is not possible to know the mol% of LPC in liposome patches once LPC is added to the experimental chamber or patch pipette because there is no possibility of controlling its incorporation and concentration in a liposome patch. LPC continues to associate with the liposome patch until the patch breaks. MscL can be fully activated by LPC as long as the LPC concentration is higher than 20 mol % ^2^.

**Figure S1.** **Patch-clamp recording from purified MscL mutant V120C reconstituted into azolectin liposomes.** Expanded views show the openings of MscL-V120C in the presence (left) and absence of suction (right) over the course of the experiment using 300 μM LPC in the patch pipette. The openings of the channel activated by LPC in the azolectin patches took longer to be observed (~37 min) when compared to the full openings recorded in POPC liposome patches in the presence of 3 and 5 μM LPC (~2 min) (**Fig. 5**). Pipette potential was +30 mV.

***Finite Element Modelling***

***Force Calculations***

By likening the forces to a nonlinear spring, the equations for a linear spring described by the Hooke’s Law, F = kx, becomes F = k/x^2^, in the case of Coulomb’s Law. The constant k is an amalgam of all the constants from the respective electrostatic force equations, as follows;

Starting with the Coulomb law:

$F=- \frac{1}{4\pi\varepsilon_{0}}\frac{q_{1}q_{2}}{r^{2}}$ (Eq. 1)

(Where *F* is the force, q1 and q2 are charge values for the interacting electrons, $\varepsilon_{0}$ is the vacuum permittivity of free space, and r is the distance between the two charges)

The distance variable is modified to (3 + r) to take into account the equilibrium position of 3 Å between the helices, so that at this position:

$F=- \frac{1}{4\pi\varepsilon_{0}}\frac{q_{1}q_{2}}{{(3+r)}^{2}}$ (Eq. 2)

In order to account for all hydrogen bonds encountered between the atoms in C-terminus residues the DSSP approximation was used. It is an algorithm which assigns secondary structure to amino acids in a protein and can be used for estimation of hydrogen bond energy^3^.

The DSSP equation is:

$E=0.084\left( \frac{1}{r_{(ON)}}+ \frac{1}{r_{(CH)}}-\frac{1}{r_{(OH)}}-\frac{1}{r_{(CN)}} \right)*332 kcal/mol$ (Eq. 3)

(where *E* is energy, r(ON) r(CH) r(OH) and r(CN) are the distances between the coordinates of the interacting backbone atoms)

Simplifying the equation by converting from kcal/mole to Joule, eliminating r(ON) r(CH) and r(CN) because only the OH backbone atoms will be modelled for the hydrogen interactions in the C-terminus, and deriving with respect to distance to find the force, yields:

$F=\left( 1.9638216e^{-9} \right)\left( -\frac{1}{{{{(2.8+r)}^{2}}_{\left( OH \right)}}_{\left( 1 \right)}} -\frac{1}{{{{(3.0+r)}^{2}}_{(OH)}}_{(2)}}-\frac{1}{(3.2+{{{r)}^{2}}_{(OH)}}_{(3)}} \right)$ (Eq. 4)

Please note that there is no generally correct H-bond definition, as there is no sharp border between the quantum-mechanical and electrostatic regimes and no discontinuity of the interaction energy as a function of distance or alignment.

To determine the electrostatic and polar forces acting in the 3D MscL FE model in an electrolyte solution the Debye-Hückel relation was used:

$E_{1,2}= \frac{q_{1}q_{2}exp(-\kappa r_{1,2})}{\varepsilon_{r}\varepsilon_{0}r_{1,2}}$ (Eq. 5)

Where $\kappa$ is the inverse of the Debye length, ε_r_ is the dielectric constant of water (78.0 at 298 K), ε_0_ is the vacuum permittivity constant, which is the absolute dielectric permittivity of the classical vacuum and r is the distance between the two charges^4^.

At room temperature (25 ^o^C) and in water for 1:1 electrolytes the following relation can be applied^5^:

$\kappa^{-1}\left( nm \right)= \frac{0.304}{\sqrt{I(M)}}$ (Eq. 6)

where κ^-1^ is expressed in nanometers and I is the ionic strength expressed in moles or mole/L. Deriving energy vs. distance, dU/dr, yields the electrostatic force taking into account the charged sites that are without a finite size embedded into the dielectric continuum of water, so that the force becomes:

$F_{1,2}=- \frac{\kappa q_{1}q_{2}\exp\left( -\kappa r_{1,2} \right)}{\varepsilon_{r}\varepsilon_{0}{(3+r)}_{1,2}} - \frac{q_{1}q_{2}exp(-\kappa r_{1,2})}{\varepsilon_{r}\varepsilon_{0}(3+{{r)}^{2}}_{1,2}}$ (Eq. 7)

**Table S1: Comparison of values from literature**[**^2^**](#_ENREF_2) **and Finite Element (FE) modelling**

|  | **Lipid bilayer thinning** | **Open state pore diameter** | **Closed state TM1 helix tilt (relative to central axis)** | **Closed state TM2 helix tilt (relative to central axis)** | **Open state TM1 helix tilt (relative to central axis)** | **Open state TM2 helix tilt (relative to central axis)** |
| --- | --- | --- | --- | --- | --- | --- |
| **Value from Literature** | 5.0 Å | 28 Å | 37.2° | 34.4° | 57.5° | 44° |
| **Computation value from FE modelling** | 4.5Å | 28Å | 39.8° | 35.9° | 57.3° | 42.2° |


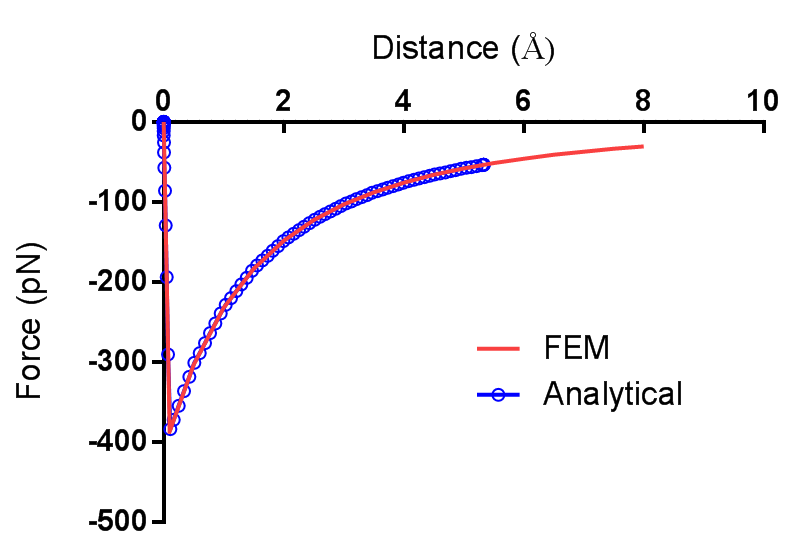


**Figure S2.** **Comparison between the first neighbour (attractive) electrostatic force.** The force was calculated for an interaction in KCl solution using the Debye-Huckel relation, and the software History output of force relative to the displacement between two helices. This simulation only contained two helices and one connector. The results were obtained using this method to implement a nonlinear force via connector elements, which match the expected values.


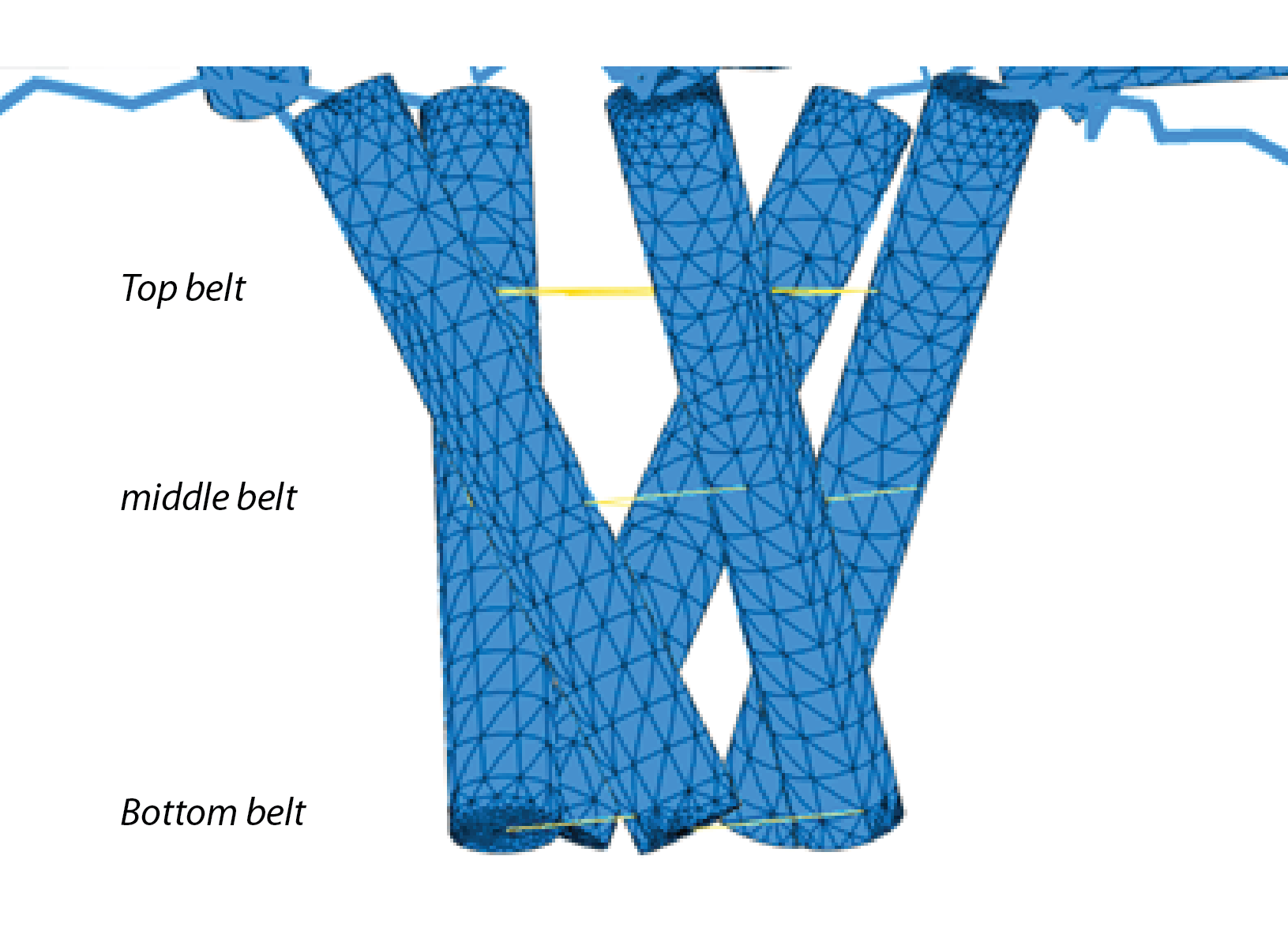


**Figure S3**. **Side view of the C-terminus structure with connectors implemented.** The three electrostatic interaction belts can be seen as thin orange lines, which are major elements of the C-terminus structure located at the top, middle and bottom of the channel. The top and bottom electrostatic interactions can be considered to be electrostatic “caps” which are stabilized by hydrogen bond networks [^6^](#_ENREF_5). The central electrostatic interactions encompass the circumference of the C-terminal bundle creating a “belt” like structure around the coiled coil. In the central belt the first neighbour interactions between the helices are attractive (opposite charge), while the second neighbour interactions are repulsive (same charge). This central belt contains an electrostatic salt bridge, which interacts with the charged RKKEE cluster [^7^](#_ENREF_6).


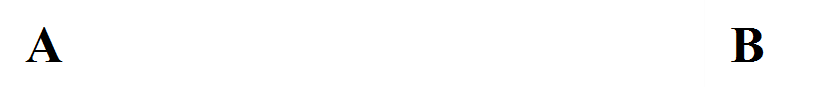


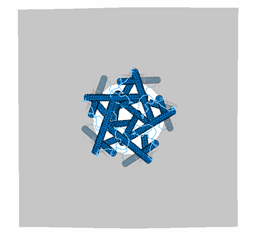

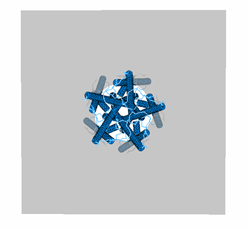

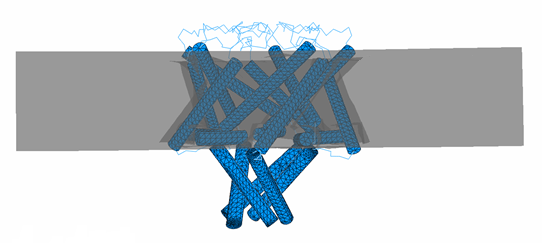

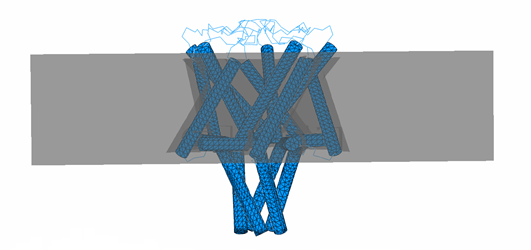
**Figure S4.** **C-terminal structural dynamics using finite element (FE) simulation for vacuum.** **(A)** Side and top views of the resting (closed) state and **(B)** open state of MscL is shown. In the open state (where the pore diameter is D ~ 30 Å and the membrane thins from 35 Å to ~ 30 Å) C-terminus has a pronounced outward bending in the upper part and lower parts of the C-terminal helix compared to the resting (equilibrated) state.


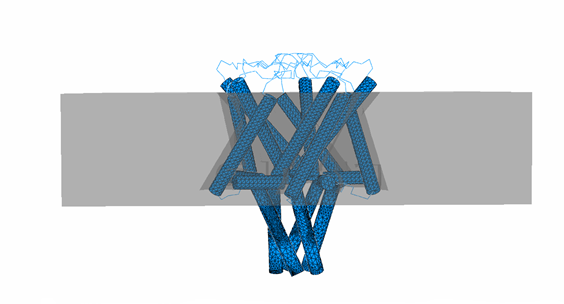

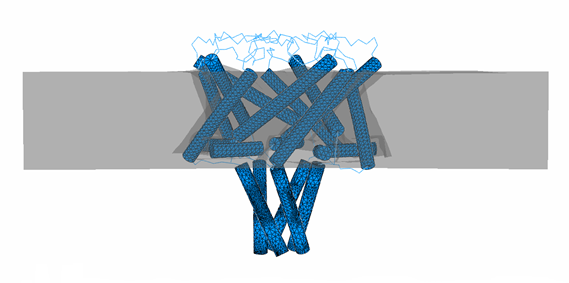


**B**

**A**


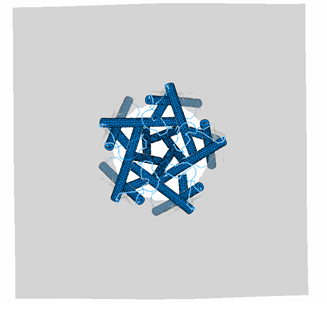

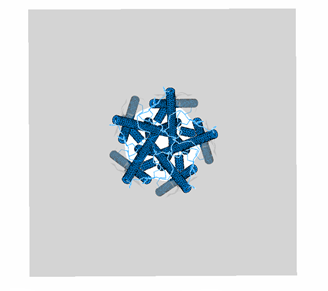
**Figure S5.** **C-terminal structural dynamics using finite element (FE) simulation for water environment.** **(A)** Side and top views of the resting (closed) state and **(B)** open state of MscL is shown. In the open state (where the pore diameter is D ~ 30 Å and the membrane thins from 35 Å to ~ 30 Å), C-terminus has a slightly visible outward bending in the upper part of the C-terminal helix compared to the resting (equilibrated) state, while the rest of the C-terminus does not change during the channel opening.

**References**

1. Nakayama, Y., Slavchov, R.I., Bavi, N. and Martinac, B. (2016) Energy of liposome patch adhesion to the pipet glass determined by confocal fluorescence microscopy. J. Phys. Chem. Letters 7: 4530−4534.

2. Perozo, E., Kloda, A., Cortes, D. M. & Martinac, B. Physical principles underlying the transduction of bilayer deformation forces during mechanosensitive channel gating. Nat Struct Biol 9, 696-703, doi:10.1038/nsb827nsb827 [pii] (2002).

3. Kabsch W, Sander C. Dictionary of protein secondary structure: pattern recognition of hydrogenbonded and geometrical features. Biopolymers. 1983; 22(12):2577±637. https://doi.org/10.1002/bip. 360221211 PMID: 6667333.

4. Ma L, Yethiraj A, Chen X, Cui Q. A Computational Framework for Mechanical Response of

Macromolecules: Application to the Salt Concentration Dependence of DNA Bendability.

Biophysical Journal. 2009;96(9):3543-3554. doi:10.1016/j.bpj.2009.01.047.

5. Israelachvili, J., Intermolecular and Surface Forces, Academic Press Inc., 1985, ISBN 0-12-

375181-0.

6. Walton, T.A. & Rees, D.C. Structure and stability of the C-terminal helical bundle of the E. coli mechanosensitive channel of large conductance. *Protein science : a publication of the Protein Society* **22**, 1592-1601 (2013).

7. Kloda, A., Ghazi, A. & Martinac, B. C-terminal charged cluster of MscL, RKKEE, functions as a pH sensor. *Biophysical journal* **90**, 1992-1998 (2006).
